# The attenuated ASFV strains MK-200 and FK-32/135 as possible models for investigation of protective immunity by ASFV infection

**DOI:** 10.1371/journal.pone.0270641

**Published:** 2022-07-07

**Authors:** Alexey D. Sereda, Anna S. Kazakova, Sanzhi G. Namsrayn, Mikhail E. Vlasov, Denis V. Kolbasov

**Affiliations:** Federal Research Center for Virology and Microbiology (FRCVM), Volginsky, Russia; Plum Island Animal Disease Center, UNITED STATES

## Abstract

African swine fever (ASF) is an infectious disease of domestic and wild pigs of all breeds and ages, with the acute form of the disease being characterized by high fever, hemorrhages in the reticuloendothelial system and a high mortality rate. Registered safe and efficacious ASF vaccines are not available. The development of experimental ASF vaccines, particularly live attenuated, have considerably intensified in the last years. There is much variability in experimental approaches undertaken by laboratories attempting to develop first generation vaccines, rendering it difficult to interpret and make comparisons across trials. ASF virus (ASFV) genotyping does not fully correlate with available cross-protection data and may be of limited value in predicting cross-protective vaccine efficacy. Recently, ASFV strains were assigned to a respective nine groups by seroimmunotype (from I to IX): *in vivo* the grouping is based on results of cross protection of pigs survived after their infection with a virulent strain (bioassay), while *in vitro* this grouping is based on hemadsorption inhibition assay (HADIA) data. Here we demonstrate the antigenic and protective properties of two attenuated ASFV strains MK200 and FK-32/135. Pronounced differences in the HADIA and in immunological test in animals allow us to consider them and the corresponding reference virulent strains of the ASFV of Mozambique-78 (seroimmunotype III, genotype V) and France-32 (seroimmunotype IV, genotype I) as useful models for studying the mechanisms of protective immunity and evaluation of the candidate vaccines.

## Introduction

African swine fever virus (ASFV) causes acute hemorrhagic fever in domestic pigs and wild boars with a mortality rate of up to 100%. Disease control is limited due to a lack of vaccines. Difficulties in the vaccine development are associated with a number of unique properties of the virus. Firstly, the virus specifically targets monocytes/macrophages, and thus aggressively intervenes in the regulation of the host’s immune response [1–3]. Secondly, the notable plasticity of the viral genome is implemented in the vector transmission of the ASFV by the *Ornithodoros* soft ticks. Lastly, the virus exhibits considerable variety in phenotypic properties, including virulence, serotypic and immunotypic characteristics, and ability to induce hemadsorption [4–14]. The development of a protective immune response occurs in the natural hosts and in domestic pigs that recover from the infection and are challenged with a homologous isolate [15]. Currently, two non-hemadsorbing strains, OURT88/3 and NH/P68, are mainly used as models for studying mechanisms of immunological protection and the development of candidate ASF vaccines [16–18].

Recently, ASFV strains were assigned into nine groups by seroimmunotype (from I to IX): *in vivo* the grouping is based on the results of cross protection of pigs inoculated by attenuated ASFV strain followed by challenge with virulent strains (immunological bioassay), while *in vitro* this grouping is based on the hemadsorption inhibition assay (HADIA) data [7–11].

The research on the development of protection against ASFV has led to the discovery of attenuated strains of first eight seroimmunotypes. Despite the fact that the selected attenuated strains met the established requirements for protection and harmlessness, they had some differences in several biological characteristics: the duration and level of viremia and the timing of the formation of virus-specific protection. The most extensive studies on the development of live vaccines against ASF were conducted with the virus of seroimmunotype IV. This was due to the fact that ASF epizootics in the 1960s–1980s in a number of European countries, in Latin America, and in the former USSR (Odessa region) were caused by viruses of seroimmunotype IV [19]. Attenuated strain FK-32/135 was selected by passaging of the virulent reference strain France-32 (seroimmunotype IV, genotype I) in porcine bone marrow cells (PBMC) culture [10, 11]. The inoculation of an attenuated FK-32/135 strain (seroimmnotype IV) at a dose of 10^4.0^ HAU_50_ created protection in pigs against other virulent ASFV isolates of seroimmunotype IV [11]. Strain FK-32/135 was distinguishable from the majority of virulent and attenuated ASFV strains by pronounced “loose” hemadsorption in PBMC and peripheral blood leukocytes of swine (PBLS) cultures.

After selective passages of Mozambique-78 (seroimmunotype III, genotype V) in the PBMC culture, the attenuated hemadsorbing strain MK-200 was obtained [10, 11]. It had moderate reactogenicity in pigs, and caused a rise in body temperature up to 40.2 °C in 20% of the pigs for 4–5 days after intramuscular administration at a dose of 10^6.0^–10^7.0^ HAU_50_. On day 14, 90% of the pigs developed resistance to infection with the parental virulent strain Mozambique-78 at a dose of 10^3.0^ HAU_50_ [11].

Here we demonstrate the antigenic and protective characteristics of two attenuated ASFV strains: MK-200 and FK-32/135. Pronounced differences in the reaction of hemadsorption delay and immunological test in animals, as well as differences in genotypes, allow us to consider them and the corresponding reference virulent strains Mozambique-78 and France-32 of ASF virus as useful models for studying the mechanisms of protective immunity and evaluation of the candidate vaccines.

## Materials and methods

### Viruses and cells

The ASFV strains were obtained from the Federal Research Center for Virology and Microbiology (FRCVM) collection of microorganisms: the virulent strains Lisbon-57 (L-57), Congo-49 (C-49), Mozambique-78 (M-78), France-32 (F-32), TSP-80, TS-7, Uganda, Stavropol 01/08, Davis; and the attenuated strains MK-200 derived from strain M-78 (seroimmunotype III, genotype V) and FK-32/135 derived from strain F-32 (seroimmunotype IV, genotype I) [7].

Infectivity of the ASFV was determined by titration in PBLS using four wells for each tenfold cultivation [20]. Results were examined by the presence of hemadsorption phenomenon after 5–7 days. The virus titers were calculated according to the method of B.A. Kerber modified by I.P. Ashmarin, and were expressed in 50% hemadsorbing units per ml (HAU_50_/mL) [21].

### Hemadsorbtion inhibition assay (HADIA)

Following reference seroimmunotype strains of ASFV were used in the experiment: I—L-57, II—C-49, III—M-78, IV—F-32, V—TSP-80, VI—TS-7, VII—Uganda, VIII—Stavropol 01/08, IX—Davis. All viral strains were refreshed (1–2 passages) in primary PBLS cells cultures and had average infectious activity of 10^6.5–7.5^ HAU_50_/mL.

The following sera were used in the experiment: serotype-specific sera of I-IX serotypes with an activity of 1:80–1:160 in HADIA, normal pig sera treated for 30 min at 56 °C.

Culture of primary PBLS cells was prepared in 48-well plastic micropanels (Nunc, Denmark) with a working volume of 1.0 mL. Wells were filled with cell suspension to achieve concentration of 3.0–3.5 million cells/mL. As a growth medium, 0.1% lactalbumin hydrolysate was used on Earle’s saline solution with 10% of donor pig blood serum, the pH of the medium was 7.60–7.65. Micropanels were placed in a CO_2_-incubator with 5% CO_2_ with a relative humidity of 90% and incubated at a temperature of 37.0±0.5 °C. After 2 days, loose cells and excess erythrocytes were removed from the micropanels. To do this, microplates were shaken for 5–15 seconds on a shaker device (6–8 vibrations/sec) and then the suspension was removed. Then, 0.9 mL of growth medium was introduced into the wells. Next, 0.05 mL of the virus with an infectious titer of 10^4.0^ HAU_50_/mL mixed with 0.05 mL of serotype-specific or normal serum were added to each well. The micropanels were placed in a CO_2_-incubator.

HADIA was performed with the following controls: cell cultures with normal pig serum to assess the quality of cell culture; serotype-specific sera of I-IX serotypes to control for the absence of nonspecific hemadsorption; ASFV of reference strains I-IX seroimmunotypes for the presence of characteristic hemadsorption.

HADIA results were taken into account after 48–72 hours with well-expressed hemadsorption (at least 2–5 cells with specific hemadsorption in the field of view of the microscope at x400 magnification) in the virus control groups and its absence in the controls of specific sera and cell culture. The delay in the hemadsorption of the studied isolate by one of the nine reference sera indicated that it belonged to the serotype of the virus for which this reference serum was obtained.

### Animal experiments and ethics statement

Both female and male pigs of a Large White breed three-four-month-old weighing 35–40 kg from the Experimental Animal Preparation Sector of the FRCVM were used. Experiments involving animals and virus were performed in accordance with the National Institutes of Health’s Guide for the Care and Use of Laboratory Animals and were approved by the institutional animal care and use and institutional biosafety committees at FRCVM. The pigs were kept and euthanized in accordance with the protocol Guide for the Care and Use of Laboratory Animals, AVMA Guidelines [22], and all efforts were made to minimize suffering.

### Design of experiment 1

The 24 pigs were divided into 3 groups of 8. Pigs in group 1, No. 1–8, were controls, they were not immunized. On the day 0 pigs in group 2, No. 9–16, were immunized intramuscularly with attenuated ASFV strain MK-200 at a dose of 10^6.0^ HAU_50_. The pigs from group 3, No. 17–24, were immunized intramuscularly with ASFV strain FK-32/135 at a dose of 10^6.0^ HAU_50_ (Table 1).

**Table 1:** Design of experiment 1.

Group No.	Pig No.	Immunization with attenuated ASFV strain on day 0		Challenge with virulent ASFV strain on day 24	
		MK-200	FK-32/135	M-78	F-32
1	1–4	-*	**-**	**+**	**-**
	5–8	**-**	**-**	**-**	**+**
2	9–12	+**	**-**	**+**	**-**
	13–16	**+**	**-**	**-**	**+**
3	17–20	**-**	**+**	**+**	**-**
	21–24	**-**	**+**	**-**	**+**

-*—did not receive injection, +*—received injection.

During the experiment on days 0, 3, 7, 14, 21 and 24 blood samples in a volume of 5 mL were collected from the cranial vena cava of pigs into test tubes with coagulant for receiving sera and with anticoagulant lithium heparin for determination of viremia levels. On the 24th day post immunization pigs No. 1–4 (group 1), 9–12 (group 2), 17–20 (group 3) were infected intramuscularly with virulent ASFV strain M-78 at a dose of 10^3,0^ HAU_50_, pigs No. 5–8 (group 1), 13–16 (group 2), 21–24 (group 3) were infected intramuscularly with virulent ASFV strain F-32 at a dose of 10^3,0^ HAU_50_. During the experiment on days 24, 27, 30, 33, 36, 39 and 42 after start of experiment the blood samples in a volume of 5 mL were taken in test tubes with anticoagulant lithium heparin for determination of viremia levels. The samples were obtained in accordance with the established rules [23].

ASFV-inoculated pigs were monitored for body temperature and other clinical symptoms.

### Design of experiment 2

Two pigs (No. 1 and 2) were intramuscularly inoculated with ASFV strain FK-32/135 at a dose of 10^6.5^ HAU_50_. Before (on day 0) and after (on day 6) the injection of the virus to the animals, 50 mL of blood was taken from each animal via jugular vein puncture. One half of the blood volume was used for serum preparation. The second half of the blood volume was mixed with heparin 20 U/mL and left for 1.5 hours at 37 °C. Then white blood was taken out and the cells were pelleted by centrifugation at 800 g for 20 min. The pellets were resuspended to a concentration of 3.0–3.5 x 10^6^ cells/mL in 0.1% lactalbumin hydrolysate on Earl’s saline solution with 10% autologous pig blood serum (PS) or 10% fetal bovine serum (FBS). Three types of suspensions of primary cell cultures were obtained: PBLS from day 0 with PS (PBLS-0-PS), PBLS from day 6 with autologous PS (PBLS-6-PS), and PBLS from day 6 with FBS (PBLS-6-FBS). Sera were added to 48-well plates (1.0 cm^3^ to each well) and cultured at 37.0 ± 0.5 °C in an atmosphere with 5% CO_2_ and at relative humidity of 90%. After 48 hours, 8 wells of each of the three cell cultures were infected with ASFV strains F-32 or M-78 at a dose of 10 HAU_50_, 8 wells from each of the cell cultures were left uninfected as controls. After another 96 hours the accumulation of ASFV in each well was determined in HADIA in culture of primary PBLS cells obtained from one intact donor.

### Detection of virus specific antibodies

Serum samples were tested using the INgezim PPA Compac solid-phase ELISA test kit (Ingenasa, Spain) in duplicate. According to the kit instructions, the status of the tested sera was expressed using coefficient of inhibition (x %).

## Results

### Immunobiological properties of attenuated ASFV strains MK-200 and FK-32/135

Virulent and attenuated ASFV strains taken after long-term storage were examined for the serotype characteristics. The results of HADIA are presented in Table 2. They confirm that strains M-78 and MK-200 belong to seroimmunotype III, and strains F-32 and FK-32/135 belong to seroimmunotype IV.

**Table 2:** Seroimmunotyping of ASFV strains.

Strains and controls	Seroimmunotype	Serotype of reference sera									Virus control
		1	2	3	4	5	6	7	8	9	
Lisbon-57 (L-57)	I	-*	+**	**+**	**+**	**+**	**+**	**+**	**+**	**+**	**+**
Congo-49 (C-49)	II	**+**	**-**	**+**	**+**	**+**	**+**	**+**	**+**	**+**	**+**
Mozambique– 78 (M-78)	III	+	+	-	+	+	+	+	+	+	+
France-32 (F-32)	IV	+	+	+	-	+	+	+	+	+	+
TSP-80	V	**+**	**+**	**+**	**+**	**-**	**+**	**+**	**+**	**+**	**+**
TS-7	VI	**+**	**+**	**+**	**+**	**+**	**-**	**+**	**+**	**+**	**+**
Uganda	VII	**+**	**+**	**+**	**+**	**+**	**+**	**-**	**+**	**+**	**+**
Rhodesia	VIII	**+**	**+**	**+**	**+**	**+**	**+**	**+**	**-**	**+**	**+**
Davis	IX	**+**	**+**	**+**	**+**	**+**	**+**	**+**	**+**	**-**	**+**
MK-200	III	+	+	-	+	+	+	+	+	+	**+**
FK-32/135	IV	+	+	+	-	+	+	+	+	+	**+**
Serum controls		-	-	-	-	-	-	-	-	-	-
Cell culture control		-	-	-	-	-	-	-	-	-	-

Note: -*—absence of cells with specific hemadsorption; +**—2–5 cells with a specific hemadsorption in the field of view of the microscope.

**Fig 1:**
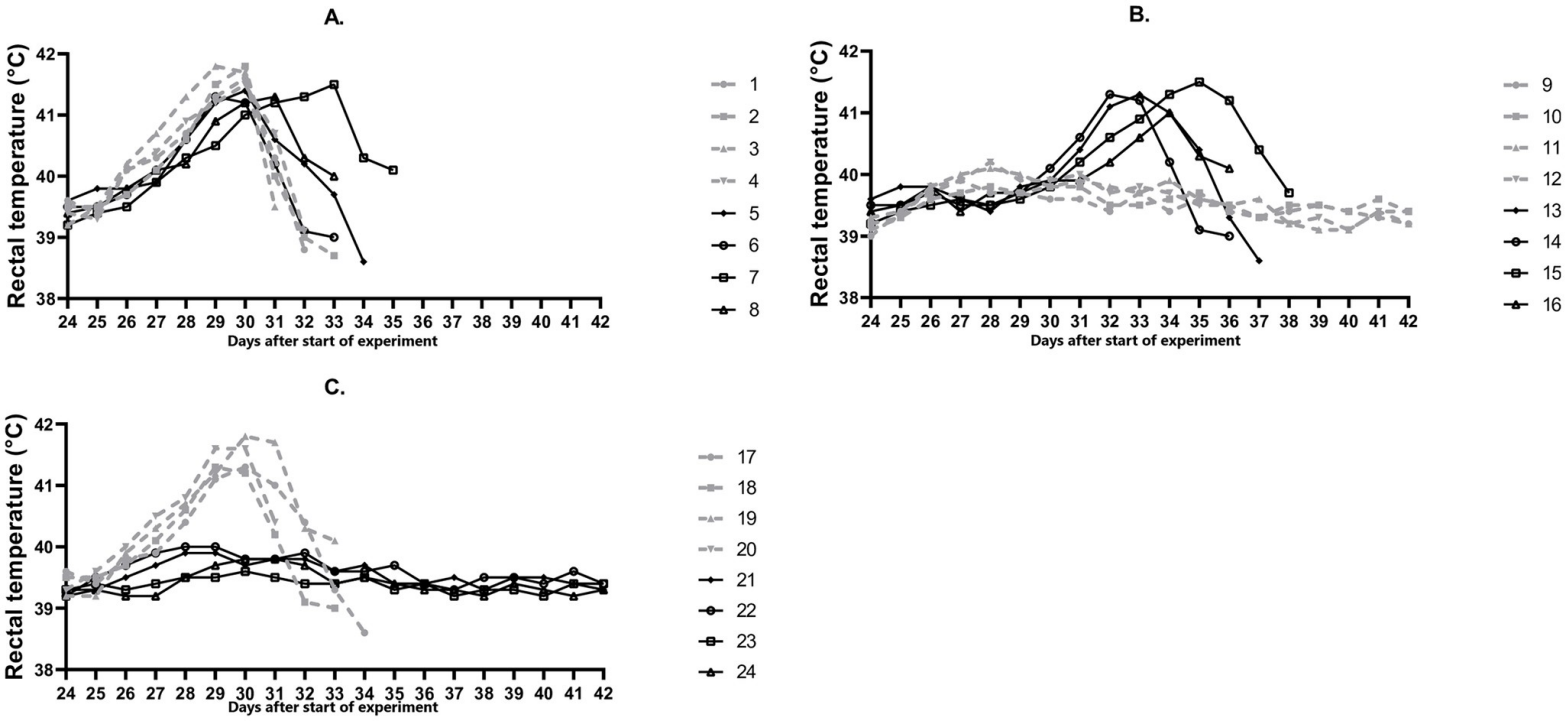
Dynamics of body temperature in animals from groups 1 (A), 2 (B), 3 (C). On day 0 pigs No. 1–8 (group 1) were not inoculated (control), No. 9–16 (group 2) were inoculated with the ASFV strain MK-200, No.17-24 (group 3) were inoculated with the ASFV strain FK-32/135. On day 24 pigs No.1-4 (A), 9–12 (B), 17–20 (C) were infected with the virulent ASFV strain M-78, pigs No. 5–8 (A), 13–16 (B), 21–24 (C) were infected with the virulent ASFV strain F-32.

**Fig 2:**
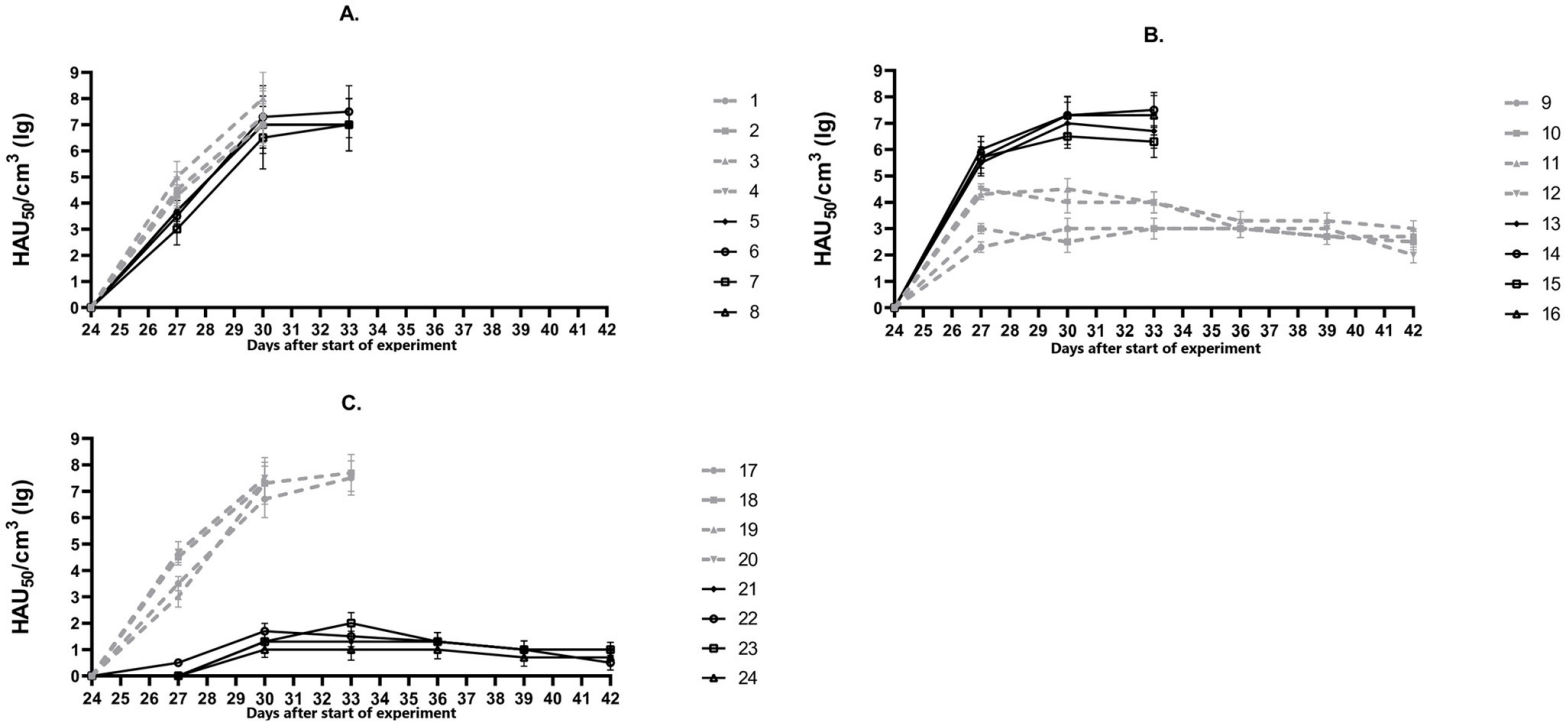
Dynamics of viremia in animals from groups 1 (A), 2 (B), 3 (C). On day 0 pigs No. 1–8 (group 1) were not inoculated (control), No. 9–16 (group 2) were inoculated with the ASFV strain MK-200, No. 17–24 (group 3) were inoculated with the ASFV strain FK-32/135. On day 24 pigs No. 1–4 (A), 9–12 (B), 17–20 (C) were infected with the virulent ASFV strain M-78, pigs No. 5–8 (A), 13–16 (B), 21–24 (C) were infected with the virulent ASFV strain F-32.

Further, ASFV strains M-78, MK-200, F-32, FK-32/135 were characterized in the immunological bioassay. As expected, pigs No. 1–4 infected with the virulent ASFV strain M-78 developed elevated temperature in 2–3 days post infection (days post challenge) (d.p.i.) (> 40 °C) (Fig 1A), then, the clinical signs started to manifest. They included: depression, lack of appetite, redness and cyanosis of the skin, diarrhea. The signs of the disease progressively worsened, the maximum values of body temperature were recorded at 6th d.p.i. and measured 41.5–41.8 °C with viremia ranging from 10^7.0^ to 10^8.0^ HAU_50_/mL (Fig 2A). The animals died from the acute form of ASF by 8-10th d.p.i. Pigs No. 5–8 infected with the virulent ASFV strain F-32 became ill starting 3-4th d.p.i (Fig 1A). Clinical signs corresponded to the acute form of ASF: depression, lack of appetite, redness and cyanosis of the skin, staggering gait. Maximum body temperature values of 41.3–41.5 °C were recorded on 5-9th d.p.i. Highest viremia was recorded on 6th d.p.i. and ranged from 10^6.5^ to 10^7.7^ HAU_50_/mL (Fig 2A). Animals died on 10-12th d.p.i.

In eight pigs, No. 9–16, inoculated with ASFV strain MK-200, no deviations from the normal clinical condition were found during the next 24 days. In the blood of these pigs from 3rd to 24th day, the ASFV was detected in titers from 10^1.3^ to 10^3.5^ HAU_50_/mL (data not shown). On day 24, four pigs from group 2, No. 9–12, were infected with the virulent ASFV strain M-78. In two of them, No. 11 and 12, a slight increase of body temperature to 40.1–40.3 °C was noted at 4th d.p.i. (Fig 1B). In all four pigs the maximum values of viremia were determined from 3rd to 9th d.p.i. (Fig 2B). From 12th to 18th d.p.i, despite viremia from 10^2.0^ to 10^3.3^ HAU_50_/mL, the clinical condition of pigs was normal, all animals survived. Four pigs from group 2, No. 13–16, infected on day 24 with the virulent ASFV strain F-32, fell ill after 3–5 d.p.i. Maximum body temperature values of 41.0–41.5 °C were recorded on 5-8th d.p.i. (Fig 1B), viremia—from 10^6.3^ to 10^7.5^ HAU_50_/mL on 6th to 9th d.p.i. (Fig 2B). The animals died from the acute form of ASF by 10-12th d.p.i. The results demonstrated the property of the attenuated ASFV strain MK-200 to protect pigs inoculated with it from getting sick and dying during subsequent challenge with the homologous by seroimmunotype virulent ASFV strain M-78, and not to protect against getting sick and dying during subsequent infection with the heterologous virulent ASFV strain F-32.

In all pigs from group 3, No. 17–24, inoculated with ASFV strain FK-32/135, no deviations from the normal clinical condition were found during the next 24 days. In the blood of these pigs, from 3rd to 10th day after inoculation, the ASFV was detected in titers from 10^0.5^ to 10^1.5^ HAU_50_/mL, from 14 to 24 days—there was no viremia (data not shown). On the 24th day after start of the experiment, four pigs from group 3, No. 17–20, were infected with the virulent ASFV strain M-78. From 3-4th d.p.i. the body temperature in these animals exceeded the norm (> 40 °C), and from 4-5th d.p.i. they developed clinical signs of acute form of ASF. The maximum values of body temperature of 41.3–41.8 °C were recorded on 5-6th d.p.i. (Fig 1C), viremia, from 10^7.3^ to 10^7.7^ HAU_50_/mL, from 6th to 9th d.p.i. (Fig 2C). On 8-11th d.p.i. animals No. 17–20 died. In pigs from group 3, No. 21–24, infected on day 24 with the virulent ASFV strain F-32, no clinical signs of the disease were found during the next 18 days, all animals survived (Fig 1C). The maximum values of the viremia, from 10^1.3^ to 10^1.7^ HAU_50_/mL, were recorded on 6th d.p.i. (Fig 2C), by 18th d.p.i viremia decreased to 100.5–10^1.0^ HAU_50_/mL. The results demonstrated the property of the attenuated ASFV strain FK-32/135 to protect pigs inoculated with it from getting sick and dying during subsequent infection with the homologous virulent ASFV strain F-32, and not to protect against getting sick and dying during subsequent challenge with the heterologous virulent ASFV strain M-78.

**Fig 3:**
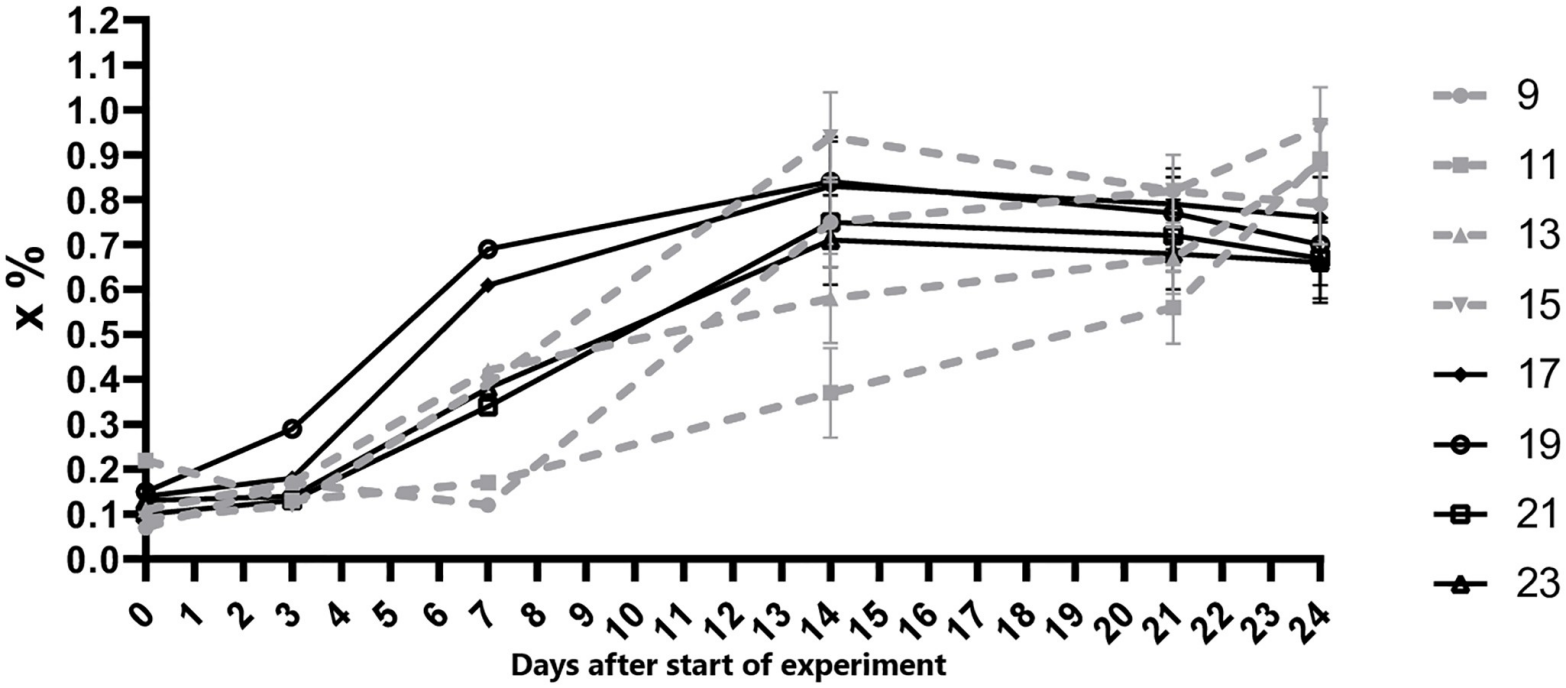
Dynamics of virus-specific antibody response in animals from the groups 2 and 3. On day 0 pigs No. 9, 11, 13, 15 (group 2) were inoculated with the attenuated ASFV strain MK-200, No. 17, 19, 21, 23 (group 3) were inoculated with the attenuated ASFV strain FK-32/135.

Samples of blood serum from pigs with odd numbers from groups No. 2 and 3 were examined for the presence of ASFV-specific antibodies. The samples obtained on days 7–14 were positive (Fig 3).

### Modeling of immune defense mechanisms *in vitro*

Previously, it was found that inoculation of the ASFV stain FK-32/135 into pigs leads to development of protective mechanisms of cellular immunity, in particular, virus-specific antibody-dependent cellular cytotoxicity (ADCC) and activity of cytotoxic T-lymphocytes (CTL), which are detected *in vitro* already on day 3 and day 6, respectively [9]. A remarkable feature of the FK-32/135 strain is the short duration of viremia [11]. This makes it possible to investigate the possibility of quantitative registration of the effects of protective immune mechanisms in ASF *in vitro* by a simple virological method. Accumulation of homologous and heterologous virulent strains of ASFV was determined in PBLS culture from pigs inoculated with FK-32/135 strain in the presence of autologous pig serum (PS) or FBS.

**Fig 4:**
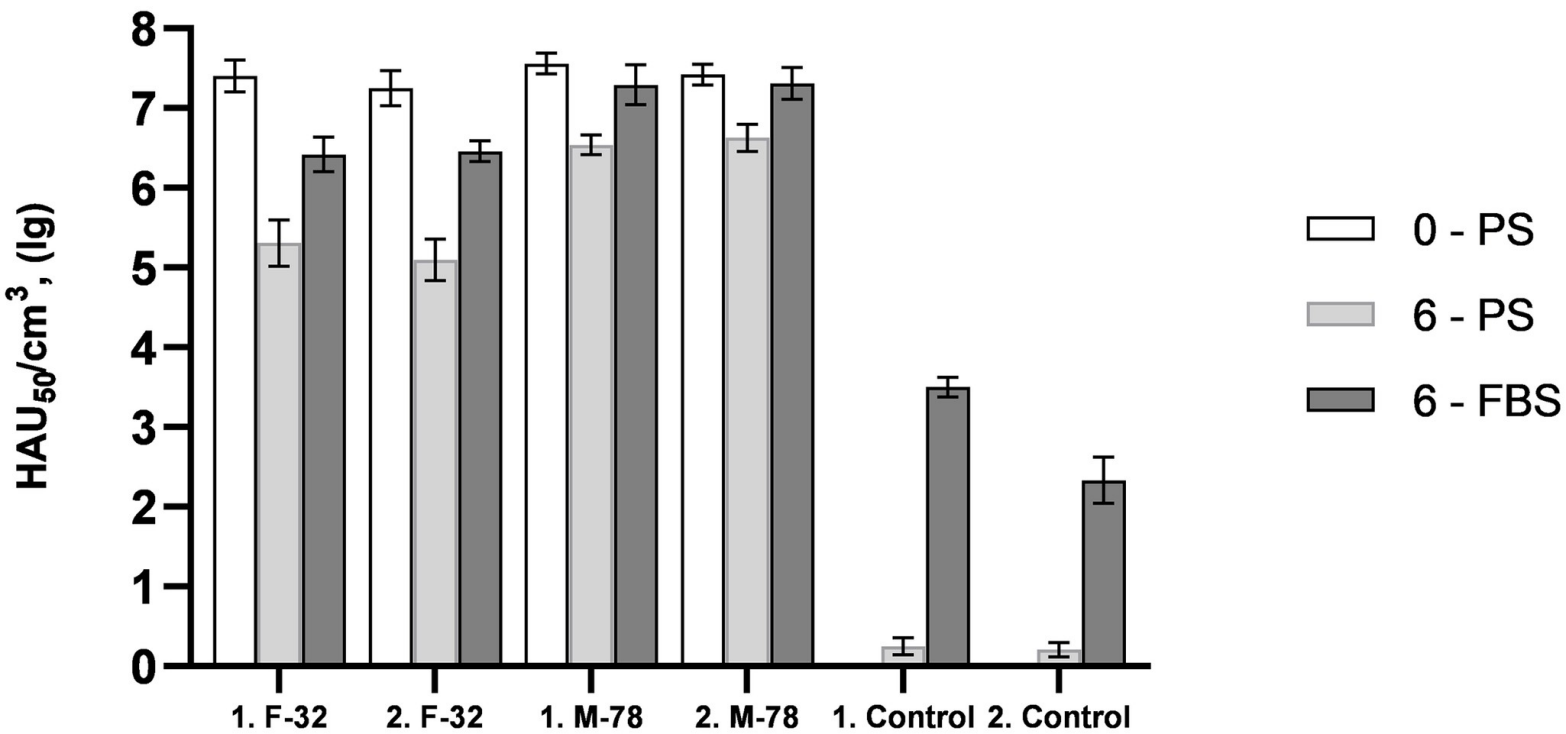
Accumulation of ASF virus strains France-32 (F-32) and Mozambique-78 (M-78) in culture of primary PBLS cells prepared from pigs No.1 and No.2 before (on day 0) and after (on day 6) inoculation of ASFV strain FK-32/135 at a dose of 10^6.5^ HAU_50_. PS—autologous pig serums, FBS—fetal bovine serum.

The results of *in vitro* modeling of the mechanisms of antiviral cellular immunity in ASF are shown in Fig 4. The accumulation of homologous ASFV strain F-32 in PBLS-6-PS compared to PBLS-0-PS was less by 10^2.09^–10^2.15^ HAU_50_/mL. The established differences are statistically significant (p<0.01). Accumulation of ASFV of heterologous strain M-78 in PBLS-6-PS compared to PBLS-0-PS was less by 10^0.79^–10^1.02^ HAU_50_/mL (p<0.01). The ASFV titers of F-32 and M-78 strains in PBLS-0-PS were similar, the differences between them were not statistically significant. In those uninfected by virulent strains of PBLS-6-PS, the accumulation of residual ASFV of strain FK-32/135 did not exceed 10^0.25^ HAU_50_/mL.

The accumulation of ASFV strain F-32 in PBLS-6-FBS compared to PBLS-6-PS was greater by 10^1.11^–10^1.36^ HAU_50_/mL (p<0.01). The corresponding differences in the accumulation of the ASFV of the M-78 strain were 10^0.68^–10^0.75^ HAU_50_/mL (p<0.01). At the same time, the accumulation of ASFV strain F-32 in PBLS-6-FBS compared to PBLS-0-PS was less by 10^0.79^–10^0.98^ HAU_50_/mL (p<0.01). The corresponding differences in the accumulation of the ASFV of the M-78 strain were 10^0.11^–10^0.27^ HAU_50_/mL (p<0.3, not statistically significant). In those uninfected by virulent strains of PBLS-6-FBS, the accumulation of residual ASFV of strain FK-32/135 reached 10^2.33^–10^3.50^ HAU_50_/mL.

## Discussion

This work confirms the importance of determining the seroimmunotype characteristics of attenuated and virulent strains in the process of development and evaluation of the candidate vaccines against ASF.

Currently, two technologies are used to categorize ASFV isolates: genotyping or seroimmunotyping. A standard methodology includes typing of the p72 capsid protein gene to provide broad inter-genotypic phylogenetic grouping with concurrent analysis of central variable region tandem repeats within the 9RL/B602L and p54/E183L genes or intergenic regions to provide intra-genotypic resolution [24–26]. To date, greater than 24 ASFV genotypes have been identified. Although useful for some purposes, ASFV genotyping does not fully correlate with available cross-protection data and may be of limited value in predicting cross-protective vaccine efficacy [27, 28].

Using HADIA and immunological bioassay, a number of researchers showed the antigenic diversity of ASFV isolates [15, 29–31]. Analysis of more than 100 virulent and attenuated strains provided the basis for ASFV seroimmunotypical classification. So far, nine seroimmunotype have been identified, but more likely exist [32, 33]. Recently, genetic signatures of serotype specificity have been identified in CD2v (EP420R, hemagglutinin) and C-type lectin-like proteins (EP153R) [34–36]. Typing in HADIA places ASFV isolates into discrete seroimmunotypes not necessarily resolved by conventional p72 genotyping. For example, ASFV of seroimmunotypes I, II and IV are all genotype I (using p72 genotype classification) [10].

With the use of seroimmunotype classification, it is possible to have a different interpretation of the results of studies in which the homo- or heterologicity of isolates was based on the genotyping of the p72 capsid protein gene or the geographical location of the virus isolation site.

Based on the results of HADIA performed using reference strains of seroimmunotypes I-IX and hemadsorption delaying type-specific porcine serum of serotypes I-IX, we confirmed that the ASFV strains M-78 and MK-200 belong to seroimmunotype III, and F-32 and FK-32/135 belong to seroimmunotype IV. Well-known fact that the pigs infected with moderately virulent ASFV or those attenuated by traditional methods develop long-term resistance to homologous, but rarely to heterologous, virus challenge [18, 37–40]. In general, the ASFV immune protection granted by ASFV live attenuated vaccines is characterized by an absence of clinical signs and by a reduction of viremia, which is either absent or delayed in onset and markedly reduced in titer [28].

In our work, inoculation of pigs with attenuated MK-200 or FK-32/135 strains did not cause clinical signs of ASF. However, the level and duration of viremia in the group of pigs inoculated with strain MK-200 were greater than in the group of pigs inoculated with strain FK-32/135. Virus-specific antibodies in pig sera were detected from day 7 after inoculation of each of the attenuated strains. In our experiments the levels of virus-specific antibodies induced by MK-200 or FK-32/135 strains did not have a statistically significant difference (p<0.05). Previously, similar results were obtained with a low-virulent ASFV isolate ASFV/NH/P68. High levels of specific antibodies against ASF were associated with chronic ASF, while low levels were observed in asymptomatic pigs after intranasal and intramuscular immunization with ASF isolate NH/P68 [41–43].

The inoculation of pigs from group 2 with attenuated ASFV strain MK-200 protected them from the disease after infection with the homologous (by seroimmunotype) virulent ASFV strain M-78. Infection of pigs from group 2 with the virulent ASFV strain F-32 led to an acute form of the disease with high temperatures and death on 8-11^th^ d.p.i. As expected, the inoculation of pigs from group 3 with attenuated ASFV strain FK-32/135 protected them from the disease after infection with the homologous (by seroimmunotype) ASFV strain F-32. The results of inoculation of animals in the group 3 in our experiments are similar to the known results with OUR T88/3 and NH/P68 isolates [27, 39]. ASFV strain FK-32/135 is interesting in its features: *in vivo* it induces asymptomatic form of ASF, and *in vitro* it induces a «loose» hemadsorption. Infection of pigs from group 3 with the heterologous by seroimmunotype virulent ASFV strain M-78 led to an acute form of the disease with high temperatures and death in 8–11 days.

To conclude, inoculation of pigs with attenuated ASFV strains MK-200 or FK-32/135 induces 100% protection against subsequent infection with homologous by seroimmunotype virulent ASFV strains M-78 or F-32, respectively. And, conversely, it does not protect animals from disease and death after infection with heterologous by seroimmunotype virulent ASFV strains F-32 or M-78, respectively.

The boundaries of homologous cross-protection are not always clear, since different ASFVs may cause cross-protection [27], and vice versa, the ASFVs that seem closely related may not provide cross-protection [39, 40]. The pairs of ASFV strains M-78 and MK-200; and F-32 and FK-32/135 are heterologous in genotypes (V and I) and in seroimmunotypes (III and IV), respectively. Whereas virulent and attenuated strains of seroimmunotypes I and II, as well as strains of seroimmunotype IV, belong to genotype I [28]. On this basis, we believe that the strains described in this paper can be used in research focused on the development of the candidate vaccines against ASF and their subsequent evaluation *in vivo*.

In experiment 2, the accumulation of homologous virulent strain F-32 in PBLS-6-PS was 100 times less than in PBLS-0-PS. Apparently, in PBLS-6-PS, at least, ADCC and CTL are functioning. The accumulation of the ASFV of the heterologous M-78 strain decreased by no more than 10 times, which may be a consequence of the action of the ADCC mechanisms. *In vitro* results confirmed that in the implementation of protection against ASF, immunological mechanisms are more effective in relation to homologous strains.

The use of fetal bovine serum in PBLS cultures, rather than autologous pig serums, made it possible to exclude the mechanism of ADCC. Therefore, in PBLS-6-FBS, the decrease in accumulation of homologous strain FK-32/135 was less pronounced compared to PBLS-6-PS, and no decrease in accumulation of heterologous strain M-78 was observed. The obtained results demonstrate the applicability of a simple virological method for preliminarily *in vitro* evaluation of ADCC and CTL mechanisms in relation to homologous and heterologous strains of the ASFV.
